# Effectiveness of virtual reality technology combined with conventional pelvic floor rehabilitation training in postpartum myofascial pelvic pain syndrome: A randomized controlled trial

**DOI:** 10.1371/journal.pone.0340918

**Published:** 2026-03-31

**Authors:** Lu Liu, Ziling Lin, Xueling Chen, Yanjun Hou, Yanping Liu, Xiangbin Wang, Meijin Hou

**Affiliations:** 1 College of Rehabilitation Medicine, Fujian University of Traditional Chinese Medicine, Fuzhou, China; 2 Key Laboratory of Orthopedics and Traumatology of Traditional Chinese Medicine and Rehabilitation Ministry of Education, Fujian University of Traditional Chinese Medicine, Fuzhou, China; 3 Grand Hospital of Shuozhou, Shuozhou City, Shanxi Province, China; 4 Department of Rehabilitation, Fuzhou Second General Hospital, Fuzhou, China; 5 The Third People’s Hospital Affiliated to Fujian University of Traditional Chinese Medicine, Fuzhou, China; 6 Rehabilitation Hospital affiliated to Fujian University of Traditional Chinese Medicine, Fuzhou, China; 7 National-Local Joint Engineering Research Center of Rehabilitation Medicine Technology, Fuzhou, China; Iran University of Medical Sciences, IRAN, ISLAMIC REPUBLIC OF

## Abstract

**Objective:**

The aim of this study was to compare the therapeutic efficacy of integrating virtual reality technology with conventional pelvic floor rehabilitation therapy versus conventional therapy alone in postpartum women with myofascial pelvic pain syndrome.

**Methods:**

Fifty-seven postpartum women diagnosed with myofascial pelvic pain syndrome were recruited for this study between March 1, 2023, and December 29, 2023. All participants were randomly assigned to two groups. The experimental group (n = 27) underwent virtual reality training combined with conventional pelvic floor rehabilitation therapy, while the control group (n = 30) received only conventional pelvic floor rehabilitation therapy. Both groups completed ten treatment sessions. Changes in pelvic floor muscle contraction function were assessed using pelvic floor surface electromyography. Musculoskeletal ultrasound was employed to measure muscle thickness and Young’s modulus of the pelvic floor muscles. The Visual Analog Scale was used to evaluate the degree of pain experienced during palpation of the pelvic floor muscles.

**Results:**

The experimental group demonstrated a significant reduction in relaxation time during the fast muscle contraction stage of the pelvic floor muscle’s Glazer S-EMG (*P* < 0.05). No statistically significant differences were observed in the Visual Analog Scale, pelvic floor muscle thickness, or Young’s modulus of the pelvic floor muscle during resting and maximum contraction states (*P* > 0.05).

**Conclusion:**

The integration of virtual reality technology with conventional pelvic floor rehabilitation therapy has the potential to improve the relaxation capacity of fast-twitch muscle fibers within the pelvic floor muscles. However, it does not seem to offer any benefits in increasing pelvic floor muscle thickness or in alleviating myofascial pelvic pain.

**Trial Registry:**

The registry and the registration number: Chinese Clinical Trial Registry (number ChiCTR2300069517).

## Introduction

Myofascial pelvic pain syndrome (MPPS) is characterized by the presence of highly sensitive trigger points within the pelvic floor muscles (PFM) and surrounding musculature. This condition manifests as pelvic floor pain accompanied by myofascial tension or spasm [1,2]. It is one of the common pelvic floor dysfunction disorders affecting women after delivery [3]. The experience of pain has been shown to cause prolonged fatigue and psychological disorders [4], which significantly affect the daily lives and occupational functioning of patients with MPPS.

Pregnancy is typically associated with significant changes in the musculoskeletal system [5]. As pregnancy progresses, the gradual increase in fetal weight leads to a corresponding rise in the load on the pelvic floor muscles (PFM) [6]. Prolonged overloading, as well as acute or repeated microtrauma can cause chronic tightening and contracture of muscle tissue [7]. This process results in local muscle hypoxia and acidosis, which gradually contribute to the formation of myofascial pain trigger points. The presence of these trigger points induce hypertonia of the PFM, leading to muscle spasms and difficulty in achieving muscle relaxation [8].

Current rehabilitation modalities include myofascial manipulation release, biofeedback therapy, electrical stimulation, and magnetic therapy, among others [9,10]. All of these treatments require a basic awareness of PFM. However, unlike other skeletal muscles, the contraction and relaxation of the PFM are not easily observable to the naked eye [11]. Previous research has shown that 70% of women with pelvic floor dysfunction are unable to correctly contract their PFM, and 97% can only achieve a weak contraction [12]. Effective contraction of the PFM is crucial for restoring its function. Therefore, it is essential to develop methods that enhance sensory perception during PFM training.

In recent years, virtual reality (VR) has been extensively utilized in managing musculoskeletal pain, emerging as an effective approach for chronic pain management [13–17]. By immersing individuals in engaging virtual environments, VR provides a multifaceted sensory experience that includes visual, auditory, and tactile stimuli [18]. Previous studies have demonstrated that VR technology can enhance muscle perception, facilitate muscle relaxation, and improve motor function [19–22]. For individuals with MPPS, impaired sensory perception and reduced relaxation capacity are critical factors that significantly influence both pain recurrence and the effectiveness of PFM training [7,23]. In clinical practice, patients with MPPS are typically instructed to perceive and relax their PFM through verbal guidance or imagery techniques. However, these methods often lack contextual and sensory intuitiveness, resulting in largely unsatisfactory outcomes. To date, no research has investigated the rehabilitative impact of VR technology combined with conventional PFM rehabilitation training for postpartum women with MPPS.

This study aimed to investigate the therapeutic potential of VR technology in enhancing PFM proprioception among individuals with MPPS. We hypothesized that VR technology would demonstrate superior efficacy in facilitating both the relaxation and contraction functions of the PFM compared to conventional rehabilitation training alone.

## Materials and methods

### Study design

This randomized controlled clinical trial recruited participants from March 1, 2023, to December 29, 2023. A total of 60 eligible subjects were enrolled and randomly assigned to either the experimental group or the control group. The study was conducted at the Third People’s Hospital and the Rehabilitation Hospital affiliated with Fujian University of Chinese Medicine. Ethical approval was granted by the Ethics Committee of the Rehabilitation Hospital affiliated with Fujian University of Chinese Medicine (approval number 2022KY-025–01. Ethical approval was obtained in written form. This study was registered on March 20, 2023, at https://www.chictr.org.cn/index.html (registration number ChiCTR2300069517). Participants who expressed willingness to participate and signed the written informed consent form were then assessed for eligibility.

### Participants

The study participants consisted of women who experienced MPPS, characterized by moderate to severe pelvic pain lasting at least six months, along with identifiable trigger points detected through palpation assessment. Inclusion criteria were as follows: (1) postpartum women meeting the MPPS diagnostic criteria [24]; (2) aged between 20 and 45 years; (3) hypertonicity of the PFM as determined by the Glazer assessment. Exclusion criteria included: (1) reproductive system-related diseases (e.g., endometriosis, acute pelvic inflammatory disease, pelvic venous congestion syndrome); (2) urinary system-related diseases (e.g., interstitial cystitis, recurrent urinary tract infection, urethral diverticulum); (3) digestive system-related diseases (e.g., irritable bowel syndrome, inflammatory bowel disease, diverticular colitis); (4) neurological disorders (e.g., brain injury, spinal cord injury, severe cognitive impairment); (5) postmenopausal women; (6) contraindications for pelvic floor ultrasonography; (7) unclean lochia; and (8) a history of pelvic girdle pain.

### Sample size

In this study, the Visual Analog Scale (VAS) was chosen as the primary measure of symptom improvement in patients with MPPS. Based on this outcome, a two-sided test with α = 0.05, a confidence level power (1-β) of = 0.8, and an expected effect size of 0.83 were established. Using G*Power 3.1 software, the required sample size for each group was calculated to be 24 participants per group. Additionally, accounting for a potential attrition rate of 20%, the total sample size required need for this study was ultimately determined to be 60 participants.

### Randomised

The project supervisor used SPSS version 25.0 to sequentially compile 60 datasets labeled 1 through 60. Random numbers were generated using the software’ s random number generator with a fixed seed value of 2,000,000. After ranking these numbers, participants were sorted in ascending order based on their ranks: those with ranked 1 to 30 were assigned to the experimental group, while those ranked 31 to 60 were allocated to the control group.

Sixty eligible MPPS participants were enrolled and assigned sequential numbers (1–60) based on their enrollment dates. The randomization sequence, generated using SPSS 25.0, was securely stored by an independent researcher who was not involved in treatment or assessment. Using this pre-generated sequence, participants were randomized into either the VR training group or the control group. Group assignments were disclosed to eligible participants by the project coordinator during their initial treatment session.

### Treatments

The control group received general pelvic floor physical therapy using the Vishee Pelvic Floor Rehabilitation Instrument (model SA9800). The therapy comprised the following components: (1) Electrical stimulation therapy, with a the frequency was set at 50 Hz, a duration of 2 seconds, and a pulse width ranging from 200 to 320 μs. The current intensity was gradually increased from 0 to the patient’s comfort threshold not exceeding 60 mA, and was applied for 10 minutes per session. (2) pelvic floor muscle training, where the training intensity was determined based on the results of the participants’ PFM evaluation results. The training intensity was set between 50% and 80% of the maximum PFM contraction strength. All participants followed to the voice-guided instructions from the biofeedback device, performing a 5-second PFM contraction followed by a 5-second relaxation phase. This cycle was repeated for 10 minutes per session. (3) Abdominal breathing exercises, conducted for 10 minutes following the voice prompts. Each treatment session lasted 30 minutes. A total of 10 treatments treatment sessions were administered, with an average frequency of 2–3 times per week.

**Fig 1:**
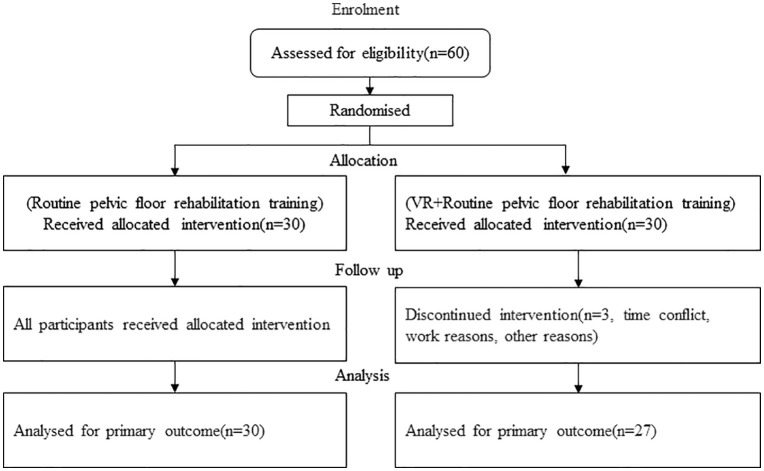
Virtual scenario of ocean.

**Fig 2:**
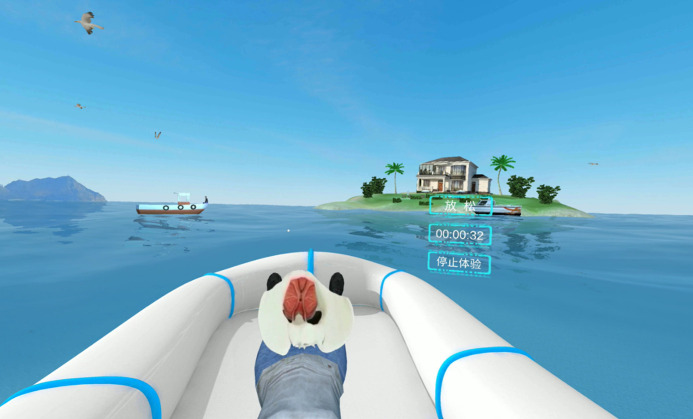
Virtual scenario of swing.

**Fig 3:**
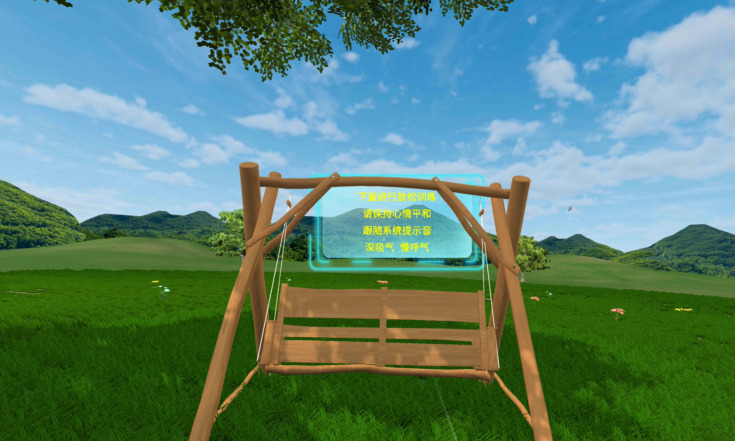
Participant flow through the stages of the randomized trial.

The experimental group received the same electrical stimulation and PFM training as the control group, with the addition of relaxation training facilitated by VR glasses (Pico 4k). In the designed simulated scenarios-Scenario 1 (Ocean, Fig 1) and Scenario 2 (Swing, Fig 2), — participants selected a scene based on their comfort level. Participants engaged in deep inhalation and slow exhalation synchronized with the rhythm of the visual display and auditory cues. During inhalation, subjects followed the guidance of a virtual character to slightly distend their abdomen while simultaneously relaxing the PFM, extending and relaxing toward the end of the body in accordance with the wave or swing movement. During exhalation, participants were instructed to slowly exhale through the mouth while contracting the abdomen and moving the PFM back and upward in harmony with the wave or swing. All treatments were administered by the same physiotherapist Fig 3.

### Outcome measurements

#### Pelvic floor muscle surface electromyography.

The Glazer assessment (Vishee, SA9800) was used to evaluate the surface electromyography (s-EMG) of the PFM in both groups before and after the intervention. The evaluation process is divided into five stages [25]: (1) Pre-resting stage: average s-EMG value (reference range: 2–4μV) and variability (reference range: < 0.2); (2) Rapid contraction stage: average s-EMG value (reference range: 35–45μV), and relaxation time (<0.5seconds). (3) Tension contraction stage: the average s-EMG value (reference range: 30–40μV), relaxation time (<1second), and variability (reference range: < 0.2); (4) Endurance contraction stage: average s-EMG value (reference range: 25–35μV), and variability (reference range: < 0.2); (5) Post-resting stage: values consistent with those observed during the preceding resting phase.

#### The thickness and Young’s modulus of the pelvic floor muscle.

The musculoskeletal ultrasound device (ACUSON Sequoia Silver) was used to measure the thickness and Young’s modulus of PFM. Muscle thickness is positively correlated with and muscle contraction function. Shear wave elastography (SWE) was employed to obtain the Young’s modulus of the PFM. The Young’s modulus value reflects muscle stiffness and tension, with higher values indicating increased tissue stiffness and muscle tension [26,27]. Following the standardized measurement position and protocol for PFM ultrasound examination [28], we assessed the thickness and Young’s modulus of the PFM at rest and during maximum contraction.

#### Pain scale.

The Visual Analogue Scale (VAS) [29] was used to assess the level of pain experienced during the palpation of the PFM, both before and after treatment.

### Follow-up

An online questionnaire survey was administered to the participants one year after the intervention concluded. The questionnaire primarily included the following items (see Table 1):

**Table 1:** The follow-up questionnaire one year after the intervention.

(1) Do you still experience MPPS?	□Yes □No
(2) If pain is present, has its intensity decreased compared to the level experienced at the end of treatment?	□Yes □No
(3) How would you describe the intensity of your pain? Please use the following scale: 0 indicates no pain, 1 to 4 represents mild pain, 5 indicates moderate pain, 6 signifies that the pain has affected your sleep, and any rating above 6 denotes severe pain.	□0 □1 □2 □3 □4 □5 □6
(4) Have you undergone any additional treatments since completing the pelvic floor rehabilitation program?	□Yes □No
(5) How would you evaluate the effectiveness of our treatment?	□very good □good □general □poor □extreme difference
(6) What was your perception of the therapy using VR glasses? (This question was directed specifically to the experimental group.)	□very good □good □general □poor □extreme difference

### Statistical analysis

The analysis was conducted using SPSS (Version 27). Data normality was assessed through visual inspection and the Shapiro-Wilk test. Continuous variables were reported as mean (SD) or median (first quartile[Q1]−third quartile[Q3]). The Student’s t-test or Mann–Whitney U test was employed to compare baseline characteristics and outcomes—including VAS, s-EMG, thickness, and Young’s modulus of the PFM—between the two groups before and after the intervention. Within-group comparisons were performed using the paired t-test. A p-value of less than 0.05 was considered statistically significant throughout the analysis. Follow-up visits with participants were conducted from December 29, 2024, to January 10, 2025.

## Results

### Baseline data of participants

A total of 60 participants were initially selected for inclusion in this study. Three were excluded from the trial group, resulting in a final sample size of 27 participants in that group. Table 2 provides a summary of the basic demographic information for both groups. No statistically significant differences were observed between the groups in terms of age, height, weight, or body mass index (BMI)(*P* > 0.05).

**Table 2:** Participants baseline data [$\overset{―}{x}$±s, M (P25, P75)].

	Experimental group (n = 27)	Control group (n = 30)	t/Z	*P*
Age (years)	31(29, 33)	32.13 ± 3.55	1.221	0.222
Height (cm)	160.85 ± 6.22	160.20 ± 5.42	0.420	0.674
Weight (kg)	56.85 ± 7.10	55.35(52.03, 60.63)	0.288	0.774
**BMI (kg/m^2^)**	22.24 ± 2.58	22.55(20.20, 24.05)	0.112	0.911

### The Glazer surface EMG of pelvic floor muscle

Following the intervention, a significant difference was observed between the two groups regarding relaxation time during the rapid contraction phase (*P* < 0.05), as shown in Table 3. The experimental group demonstrated a shorter recovery period from maximum contraction to the resting state.

**Table 3:** Comparison of pelvic floor muscle s-EMG between two groups after intervention [$\overset{―}{x}$±*s*, M (P25, P75)].

Stage	Experimental group (n = 27)	Control group (n = 30)	t/Z	*P*
Mean of pre-resting (μV)	2.87(1.54, 4.49)	3.32(2.04, 5.71)	0.543	0.587
Maximum value of the rapid contraction (μV)	42.38 ± 12.41	37.23 ± 14.17	1.460	0.151
Relaxation time of rapid contraction (s)	0.77(0.70, 0.91)	0.94(0.8, 1.19)	2.966	**0.003**
Mean value of tension contraction (μV)	36.54 ± 14.28	31.20 ± 11.79	1.550	0.128
Coefficient of variation during tension contraction	0.25 ± 0.04	0.24 ± 0.05	0.910	0.367
Relaxation time of tension contraction (s)	0.7(0.59, 0.86)	0.87(0.66, 1.27)	1.607	0.108
Mean of the contraction phase of endurance (μV)	33.66(21.67, 44.17)	27.54 ± 9.98	1.814	0.070
Coefficient of variation during endurance contraction	0.17(0.15, 0.21)	0.18 ± 0.05	0.497	0.619
Mean of post-resting (μV)	5.23(3.67, 8.02)	6.33 ± 3.21	0.503	0.615

### Thickness and young’s modulus of the pelvic floor muscle

Table 4 shows the differences in musculoskeletal ultrasound measurements of PFM between the two groups. There were no statistically significant differences in the thickness or Young’s modulus of the PFM at both the resting state and maximum contraction state following the intervention (*P* > 0.05).

**Table 4:** Comparison of thickness and Young’ s modulus of pelvic floor muscle between two groups after intervention [$\overset{―}{x}$±*s*, M (P25, P75)].

	Stage	Experimental group (n = 27)	Control group (n = 30)	t/Z	*P*
Thickness	Rest (cm)	0.89 ± 0.24	0.89 ± 0.20	−0.060	0.949
	Maximum contraction (cm)	0.92 ± 0.25	0.95 ± 0.21	−0.440	0.663
Young's modulus	Rest (kPa)	5.83(4.60, 9.33)	6.27(4.86, 8.28)	0.376	0.707
	Maximum contraction (kPa)	13.87(10.53, 23.77)	14.51 ± 6.70	0.831	0.406

### Visual Analog Scale (VAS)

**Fig 4:**
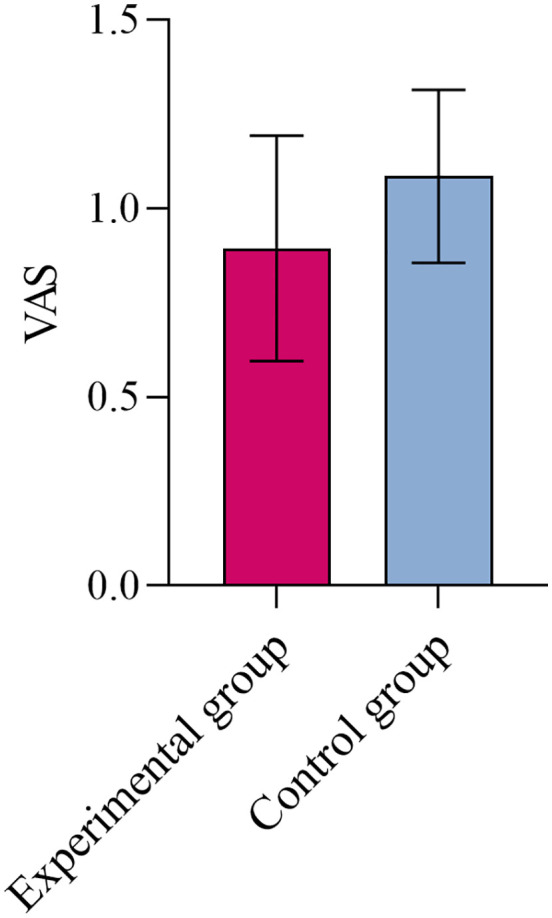
Comparison of VAS results between two groups after intervention.

**Fig 5:**
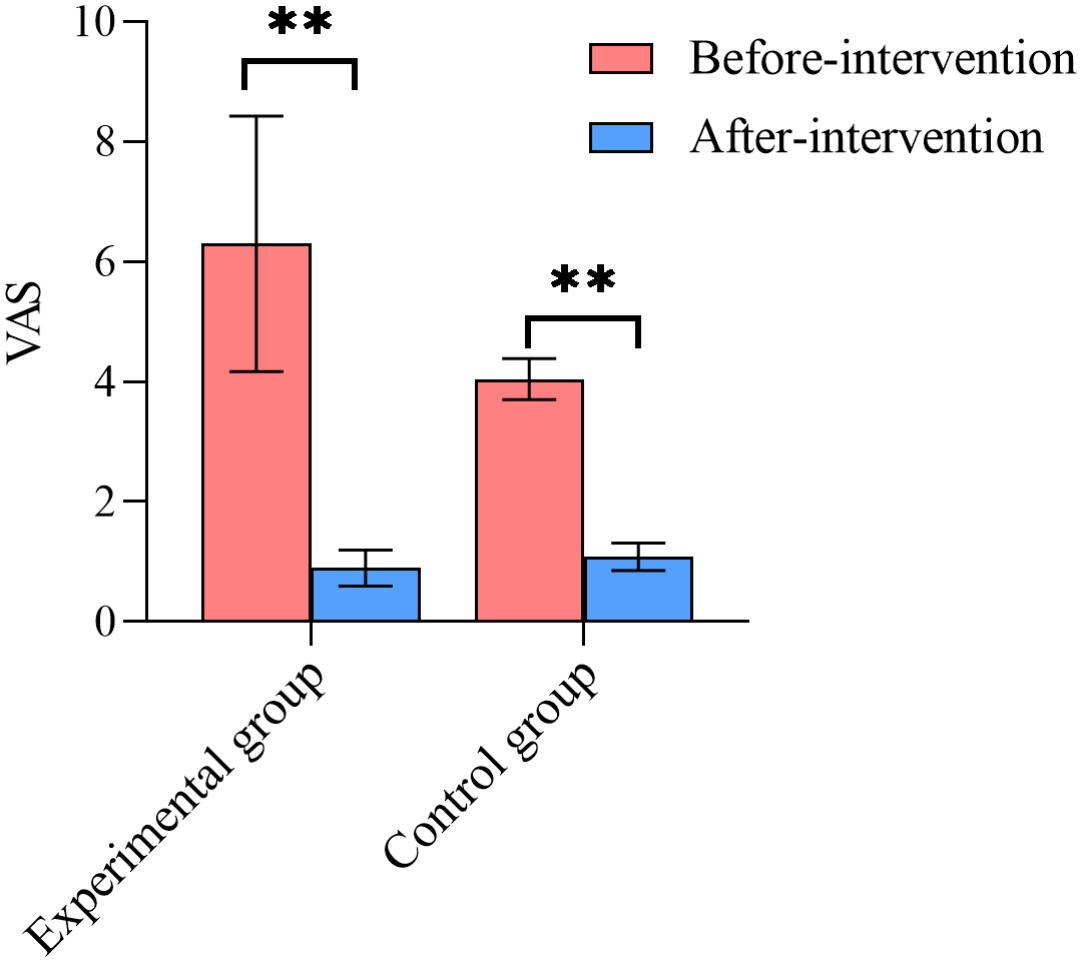
Intra-group comparison of VAS of the two groups.

Fig 4 shows the comparison of VAS scores between the two groups. There was no statistically significant difference in VAS scores after the intervention (*P* > 0.05). However, both the experimental and control groups demonstrated a significant reduction in VAS scores following the intervention compared to pre-treatment levels (*P* < 0.05), as illustrated in Fig 5.

### Follow up results

**Fig 6:**
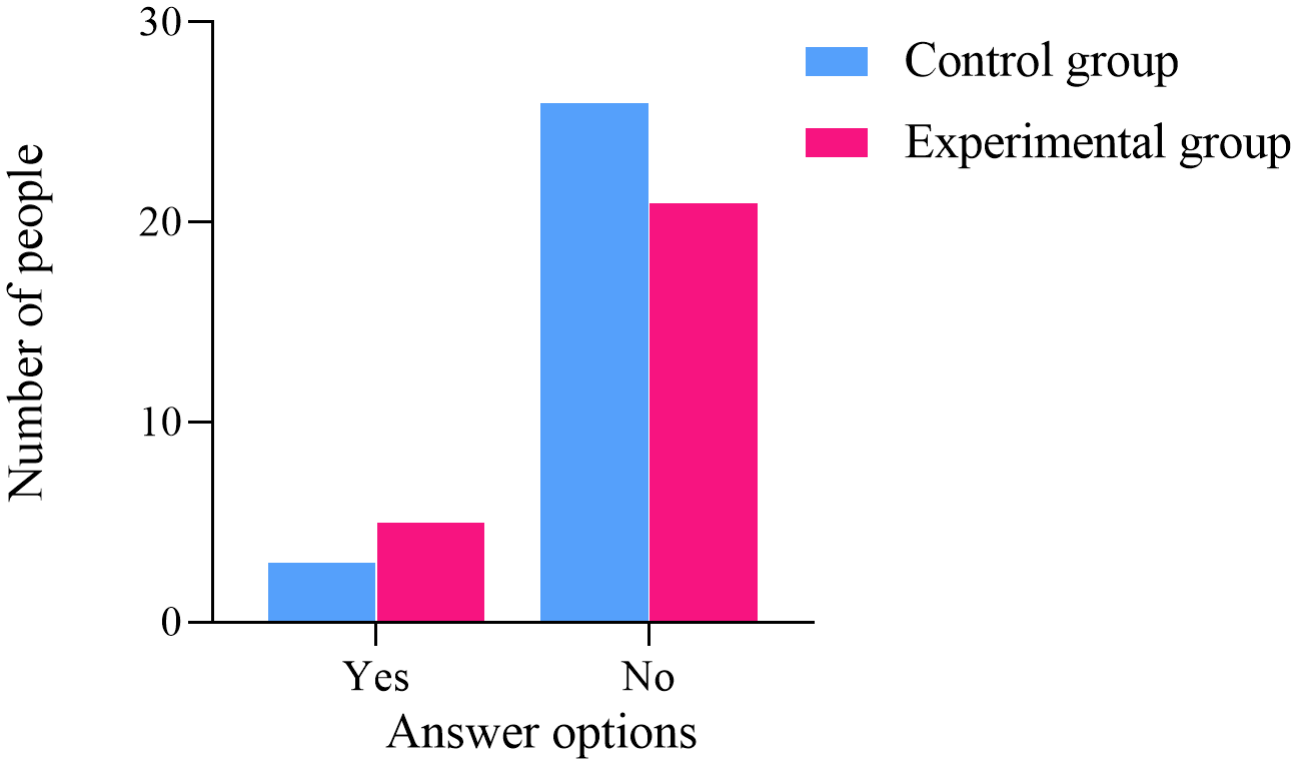
Question 1.

**Fig 7:**
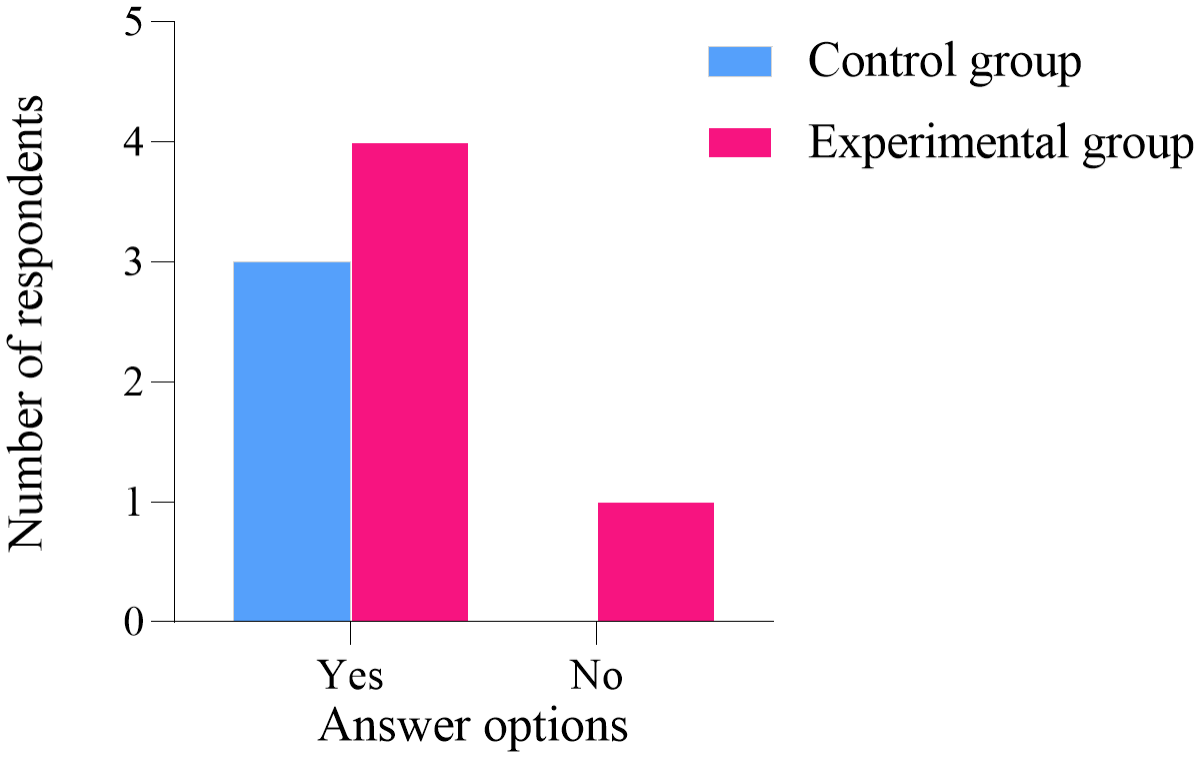
Question 2.

**Fig 8:**
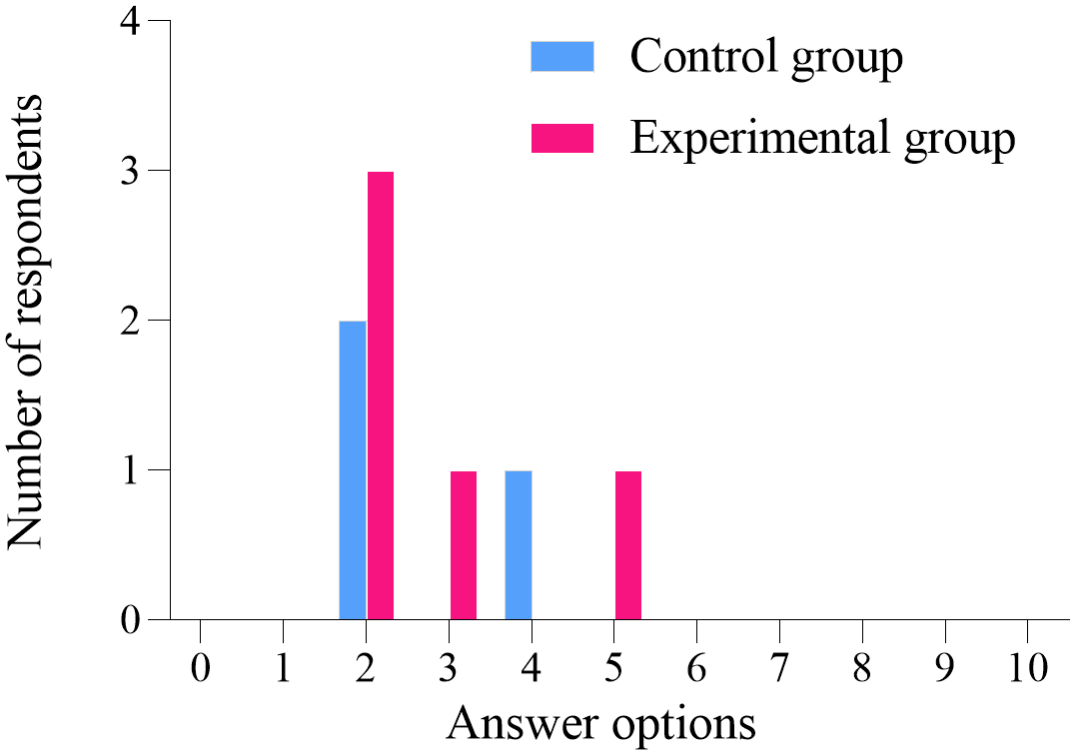
Question 3.

**Fig 9:**
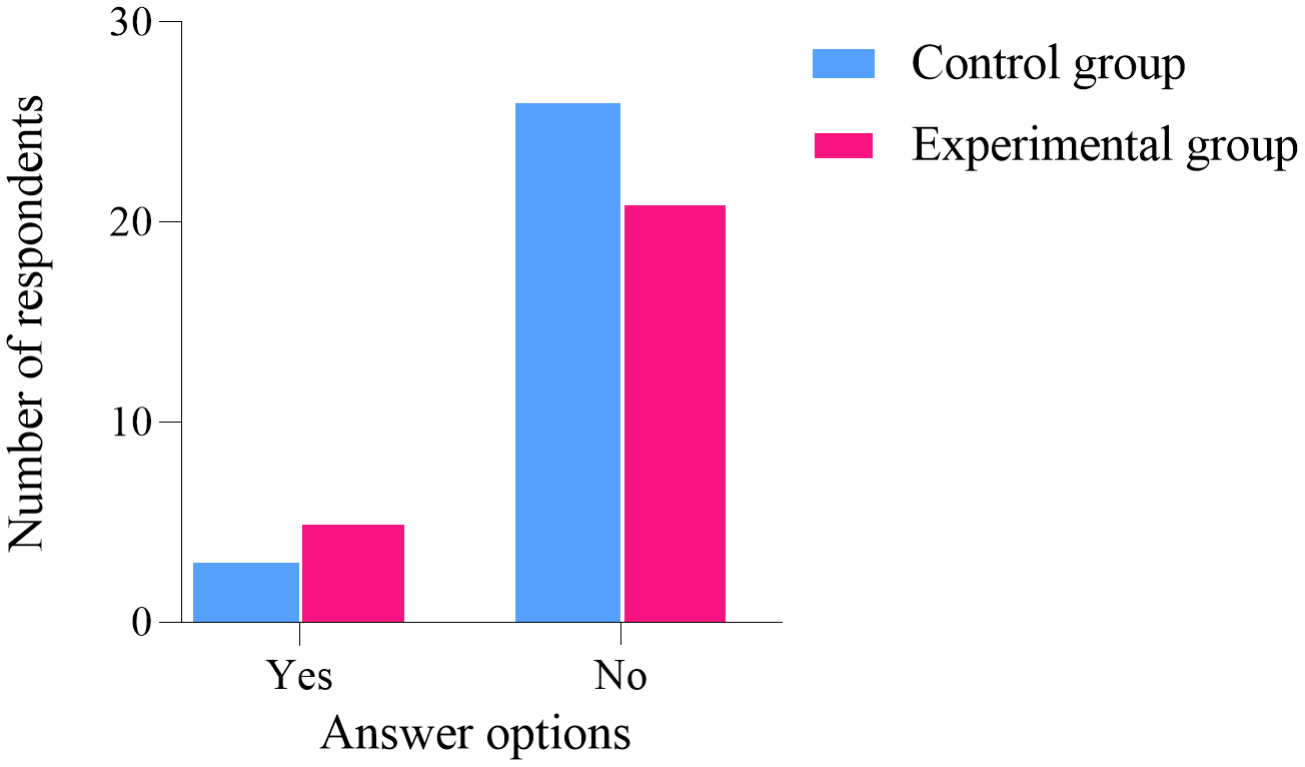
Question 4.

**Fig 10:**
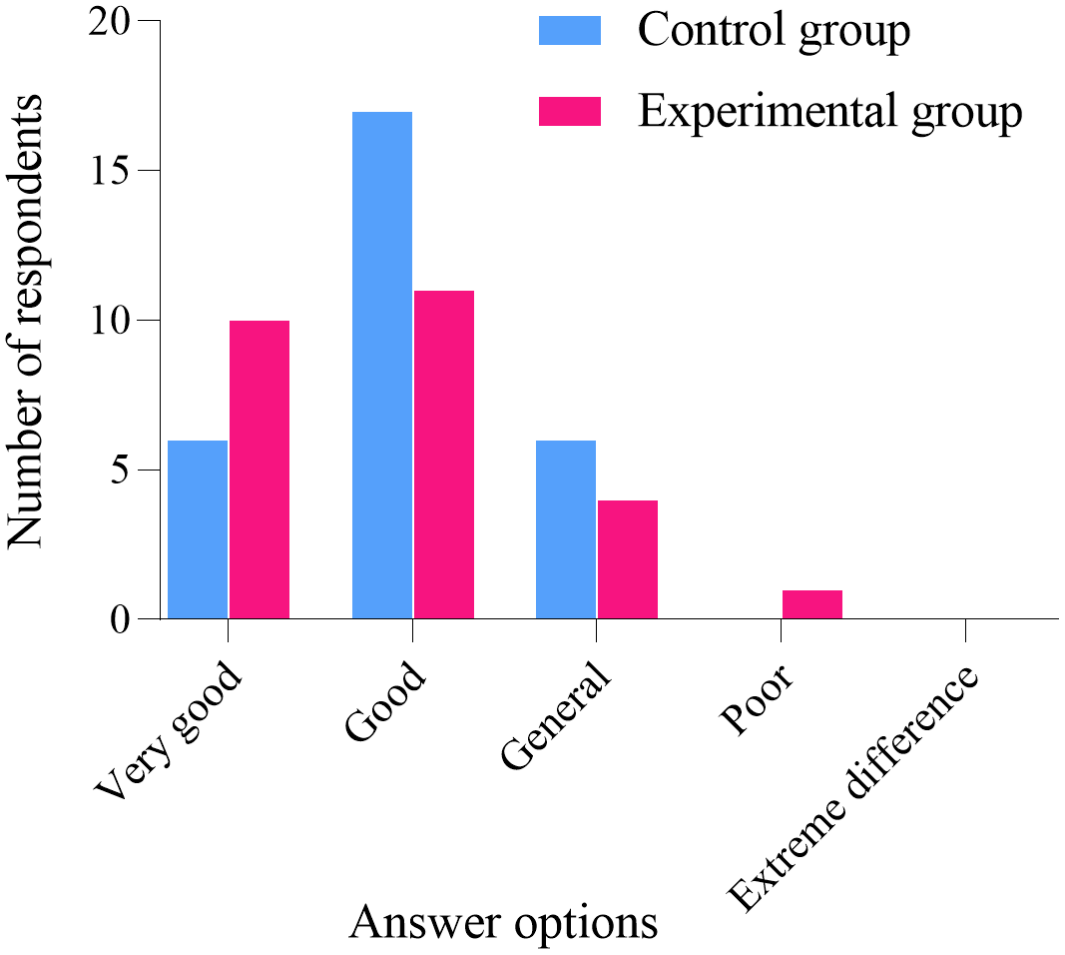
Question 5.

**Fig 11:**
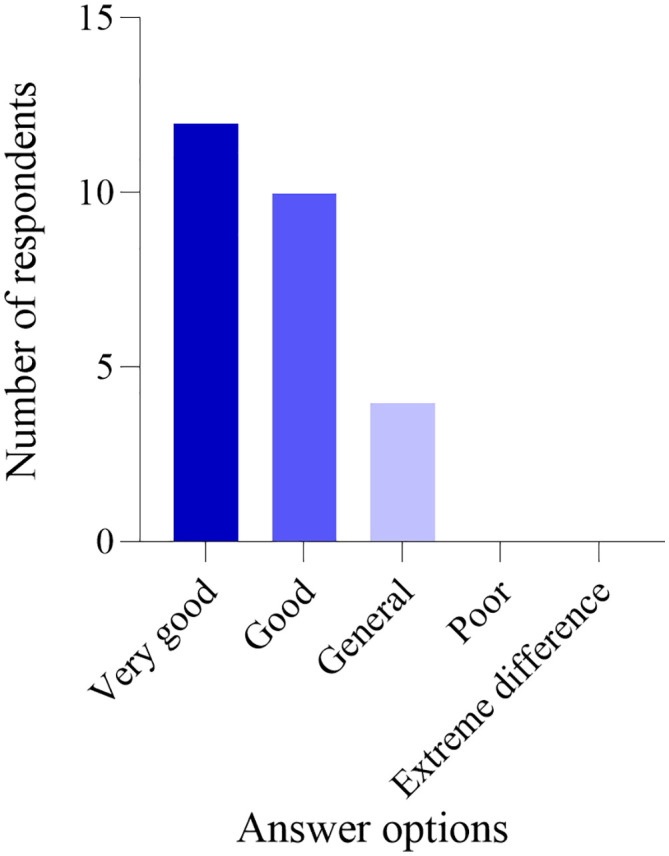
Question 6.

One year after the conclusion of the project intervention, an online questionnaire survey was conducted among the participants. A total of 26 women from the experimental group participated, while one woman did not. Similarly, 29 women from the control group participated, with one woman also not participating. The statistical analysis of the five follow-up questions showed no significant difference between the two groups (*P* > 0.05), as illustrated in Figs 6–10. Finally, the perceptions and experiences of the participants in the experimental group following VR therapy are presented in Fig 11.

## Discussion

The objective of this study was to evaluate the efficacy of VR technology combined with general pelvic floor rehabilitation therapy compared to general pelvic floor rehabilitation alone. Following the intervention, the relaxation time during the rapid contraction stage was significantly shorter in the trial group. These results indicate an improved ability to relax fast-twitch muscle fibers, suggesting that the integration of VR has a more pronounced effect on enhancing the relaxation capacity of PFM.

### Pelvic floor muscle s-EMG

Our findings revealed that the relaxation time of the fast muscle in the experimental group was significantly shorter than that in the control group. This result indicates that the relaxation ability of PFM in the experimental group was significantly improved compared to the control group. To our knowledge, individuals with MPPS exhibit PFM overactivity and hypertonicity, which impede PFM relaxation and result in prolonged relaxation time [30]. These results are consistent with the findings of Yongmei Shi [31], who demonstrated that PFM exercises based on VR technology can significantly improve PFM tone among individuals with postpartum pelvic floor dysfunction.

There may be three reasons for the significant differences observed in fast muscle function. First, previous studies have identified a correlation between chronic pain and muscle tension [32]. The immersive nature of VR promotes muscle relaxation, potentially alleviating overactivity in these muscles [33–35]. Second, anxiety symptoms are commonly observed in individuals with MPPS [36]. Numerous studies have demonstrated that VR can effectively reduce stress, anxiety, and pain [34,37,38], which may contribute to the relaxation of the PFM. Third, VR technology engages multiple senses, including visual and auditory perception, thereby stimulating the brain in a multisensory manner [39]. Previous research has shown that the immersive experience of VR can evoke a profound sense of presence, enhancing the efficacy of motor training through the mediation of mirror neurons [40,41]. In summary, our findings suggest that VR may facilitate relaxation of the PFM and relieve tension in these muscles.

### The thickness of the pelvic floor muscle

The results of this study indicate that there are no significant differences in the thickness and Young’s modulus of the PFM between the two groups. Our findings are consistent with those of two previous studies. Cho et al. reported that a VR-based running training program did not effectively increase the thickness of the non-paralyzed side of the medial gastrocnemius after six weeks [42]. Similarly, Gopal Nambi et al. [43] found no statistically significant change in lumbar multifidus muscle thickness among individuals with chronic non-specific low back pain following the VR intervention. Although VR training is insufficient to alter muscle thickness, it can effectively stimulate sensory receptors, thereby enhancing muscle strength and motor function [43]. Studies have confirmed that VR, as a modality for pelvic floor muscle training, can improve participant compliance, alleviate voiding symptoms, and enhance PFM function. It is an important adjunctive therapy for PFM rehabilitation in patients with urinary incontinence [42]. In summary, VR can serve as an auxiliary method to enhance the contractile function of the PFM, but changes in muscle thickness may require long-term intervention.

### VAS

The study observed a reduction in VAS scores among participants in both groups. However, no significant difference was found between the groups, indicating that the two intervention methods had similar effects in alleviating pain. This result aligns with previous studies [44,45], which also reported no significant differences in VAS scores when comparing VR interventions with conventional training in individuals suffering from neck pain or non-specific chronic low back pain. Nevertheless, the findings regarding the effect of VR on chronic pain remain inconsistent. A meta-analysis [46] examining the impact of VR on acute and chronic pain demonstrated that VR is an effective tool for alleviating acute pain, while maintaining long-term analgesic effects for chronic pain remains challenging. This inconsistency may be attributed to factors such as the characteristics of the VR device and the dosage of the intervention. An inadequate intervention dosage is likely to result in no significant alleviation of chronic pain. Furthermore, the primary symptom of MPPS is pain caused by the presence of myofascial trigger points. It is possible that the VR intervention was insufficient in addressing these trigger points, which may explain the lack of significant differences in VAS scores between the two groups.

### Follow-up results

One year after the conclusion of the intervention, an online questionnaire survey was conducted among the participants. A total of 26 individuals from the experimental group participated, with one individual not participating; similarly, 29 individuals from the control group participated, also with one individual not participating. The survey results indicate that 80.77% of participants in the experimental group experienced complete resolution of their pain, and 79.31% expressed a favorable perception of the treatment using VR glasses. These findings suggest that VR intervention produces positive outcomes and may serve as a potentially viable alternative to conventional pelvic floor rehabilitation training.

## Conclusions

Virtual reality can enhance the relaxation capacity of fast-twitch muscle fibers in the pelvic floor muscles, thereby improving their motor function. However, it does not increase muscle thickness or reduce muscle pain.

### Study limitations

This study has several limitations that should be acknowledged. First, the design included only two VR scenes, resulting in a limited number of training scenarios for participants and making it difficult to accommodate diverse participant preferences. Second, all participants were diagnosed with mild MPPS, which limited the ability to demonstrate significant efficacy. Third, the sample size was relatively small. Additionally, MPPS is a complex multisystem disorder, and the efficacy achieved through VR alone may be insufficient. Therefore, a multidisciplinary and multi-technological approach is essential.

## Supporting information

S1 File CONSORT checklist. (DOCX)

S2 File Clinical Research Protocol. (DOCX)

S3 File Participants baseline data. (XLSX)

S4 File The thickness and Youngs modulus of pelvic floor muscle. (XLSX)

S5 File VAS. (XLSX)

S6 File Pelvic floor muscle surface electromyography. (XLSX)

S7 File Question 1-Fllow-up questionnaire. (XLSX)

S8 File Question 2-Fllow-up questionnaire. (XLSX)

S9 File Question 3-Fllow-up questionnaire. (XLSX)

S10 File Question 4-Fllow-up questionnaire. (XLSX)

S11 File Question 5-Fllow-up questionnaire. (XLSX)

S12 File Question 6-Fllow-up questionnaire. (XLSX)
