# Supplementary material for: Effectiveness of virtual reality technology combined with conventional pelvic floor rehabilitation training in postpartum myofascial pelvic pain syndrome: A randomized controlled trial
Source: PLoS One. 2026 Mar 31;21(3):e0340918. doi: 10.1371/journal.pone.0340918 (PMC13038010; doi:10.1371/journal.pone.0340918)
Supplement: S2 File — (DOCX) [file pone.0340918.s002.docx]

**Clinical Research Protocol**

**1. Research Background**

Myofascial pelvic pain syndrome (MPPS) is characterized by the presence of highly sensitive trigger points within the pelvic floor muscle (PFM) and surrounding musculature. This condition manifests as pelvic floor pain accompanied by myofascial tension or spasm. It is one of the common pelvic floor dysfunction diseases disturbing women after delivery. The experience of pain has been shown to lead to prolonged fatigue and psychological disorders, which significantly impact the daily life and occupational functioning of patients with MPPS.

Prolonged overloading and acute or repeated micro-trauma can lead to chronic tightening and contracture of muscle tissue. This results in local muscle hypoxia and acidosis, gradually forming myofascial pain trigger points. The presence of these myofascial pain trigger points leads to hypertonia of the PFM, causing muscle spasms and making relaxation difficult.

The current rehabilitation modalities include myofascial manipulation release, biofeedback therapy, electrical stimulation, and magnetic therapy, among others. All of these treatments require a basic perception of PFM. However, unlike other skeletal muscles, the contraction and relaxation of the PFM are not easily noticeable to the naked eye. Previous research has shown that 70% of women with pelvic floor dysfunction are unable to correctly contract their PFM, and 97% of women can only achieve a weak contraction. Effective contraction of the PFM is crucial for rehabilitating its function. Therefore, it is essential to develop methods that enhance sensory perception during PFM training.

Previous studies showed that VR technology can enhance muscle perception, facilitate muscle relaxation, and improve motor functions. For individuals with MPPS, impaired sensory perception and reduced relaxation capacity are crucial factors that significantly affect both pain recurrence and the effectiveness of PFM training. In clinical practice, patients with MPPS are typically instructed to perceive and relax their PFM through verbal guidance or imagery techniques. However, these methods often lack contextual and sensory intuitiveness, resulting in largely unsatisfactory outcomes. To date, no research has investigated the rehabilitative impact of VR technology in conjunction with conventional PFM rehabilitation training for postpartum women with MPPS.

This study aimed to investigate the therapeutic potential of VR technology in enhancing PFM proprioception among individuals with MPPS. We hypothesized that VR technology would demonstrate superior efficacy in facilitating both the relaxation and contraction functions of the PFM compared to conventional rehabilitation training alone.

**2. Subject Recruitment**

Eligible subjects will be recruited from the Affiliated Rehabilitation Hospital and the Affiliated Third People's Hospital of Fujian University of Traditional Chinese Medicine. A total of 60 qualified subjects were recruited and randomly divided into the experimental group and the control group.

**Inclusion Criteria for MPPS group**

(1) postpartum women meeting the MPPS diagnostic criteria;

(2) aged ranging from 20 to 45 years;

(3) hypertonicity of the PFM as determined by the Glazer assessment.

**Exclusion Criteria**

(1) reproductive system-related diseases (e.g., endometriosis, acute pelvic inflammatory disease, pelvic venous congestion syndrome);

(2) urinary system-related diseases (e.g., interstitial cystitis, recurrent urinary tract infection, urethral diverticulum);

(3) digestive system-related diseases (e.g., irritable bowel syndrome, inflammatory bowel disease, diverticular colitis);

(4) neurological disorders (e.g., brain injury, spinal cord injury, severe cognitive impairment);

(5) postmenopausal women;

(6) contraindications for pelvic floor ultrasonography;

(7) unclean lochia;

(8) a history of pelvic girdle pain.

**Withdrawal/Exclusion Criteria**

Subjects who meet inclusion criteria, sign the informed consent form, but discontinue participation prematurely (voluntarily or involuntarily) due to the following reasons will be considered withdrawn or excluded:

(1) Misenrollment due to non-compliance with inclusion criteria;

(2) Incomplete clinical data affecting safety assessment;

(3) Refusal to undergo required evaluations;

(4) Voluntary withdrawal by the subject;

(5) Severe adverse events (e.g., falls, allergies);

(6) Other circumstances deemed appropriate for termination by researchers.

Researchers must document the reasons for termination and record outcome indicators at the time of withdrawal, especially for subjects who voluntarily withdraw.

**3. Outcome Measures and Assessment Methods**

**3.1 Pelvic floor muscle surface electromyography**

In this study, the Myotac-Pro biofeedback device (SM9800, Canada) was utilized for Glazer surface electromyography (sEMG) assessment of the pelvic floor muscles.

Prior to assessment, participants were instructed to empty their bladders and positioned according to a standardized clinical protocol: semi-supine position with the torso inclined at approximately 120 degrees relative to the lower body, legs extended naturally, hips externally rotated, and heels slightly abducted to minimize interference from adductor muscle activity on pelvic floor EMG signals.

With the participant in a fully relaxed state, the assessor gently inserted a vaginal electrode, ensuring bilateral metal plates were positioned adjacent to the levator ani muscles. Surface electromyography (sEMG) signals were recorded from the pelvic floor, while concurrent abdominal electrodes were placed to monitor abdominal muscle activation.

The assessment protocol comprised four sequential phases:

**①**Pre-resting stage**:** Participants were instructed to fully relax their pelvic floor muscles for a duration of 60 seconds to record baseline electromyographic activity and assess resting muscle tone. Normal values for mean resting potential were established at 2–4 μV, with a coefficient of variation <0.2.

**②**Rapid contraction stage**:** It included the detection of 5 rapid contractions, with a primary focus on the maximum electromyographic value and relaxation time of fast-twitch muscle fibers (i.e., type II muscle fibers) during rapid contractions. The muscle strength and relaxation ability of fast-twitch muscles were evaluated based on these data.The average s-EMG value (reference range: 35 - 45μV), and relaxation time (<0.5 seconds).

**③**Tension contraction stage**:** The subjects were required to complete 5 cycles of contraction and relaxation movements, with each contraction lasting 10 seconds and each relaxation lasting 10 seconds. The electromyographic values, coefficient of variation, and relaxation time of slow-twitch muscle fibers (type I muscle fibers) were assessed to reflect the muscle strength and coordination of slow-twitch muscles.

**④**Endurance contraction stage**：**A 60-second continuous contraction was performed, and the average electromyographic value and variability of slow-twitch muscle fibers within 60 seconds were evaluated to reflect the endurance of slow-twitch muscles.

**⑤**Post-resting stage**：**The resting electromyographic signals of muscles in a relaxed state after contraction were evaluated. The values are consistent with those observed during the preceding resting phase.

**3.2The thickness and Young's modulus of pelvic floor muscle**

Prior to the test, the materials required for the test (disposable examination gloves, disposable bed sheets, ultrasonic probe isolation acoustic transmission membranes, and ultrasonic coupling agent) were prepared. The participants were instructed to empty their bladder and bowels, then lie supine on the examination bed, relax their entire body, flex their hips and knees of both lower limbs, separate their knees, and expose the perineum. The operator sat on the left side of the subject to operate the instrument. A 5C1 probe was selected, over which a layer of ultrasonic isolation acoustic transmission membrane was applied, followed by the application of coupling agent. The specific measurement methods are as follows:

**3.2.1The thickness of pelvic floor muscle**

Referring to the standard measurement position and protocol for pelvic floor ultrasound examination, the probe was placed transversely on the perineum with its indicator pointing to the right side of the subject, and the probe was approximately at a 45-degree angle to the horizontal plane to obtain the image of the pelvic floor muscles. The "Distance" function was used to measure the thickness of the pelvic floor muscles in the resting state and during maximum contraction (the key to an effective maximum contraction is to promote the cephaloventral movement of pelvic organs while reducing the area of the levator ani hiatus).

**3.2.2 The Young's modulus of pelvic floor muscle**

①Young's modulus value at rest: With the participant's pelvic floor muscles relaxed, the operator selected the "VT" function and switched to the Young's modulus measurement module. After identifying the pelvic floor muscles, the size of the region of interest (ROI) box was adjusted, and "Update" was clicked to generate an elastogram. Three positions within the region were selected for measurement, as shown in Figure 1.


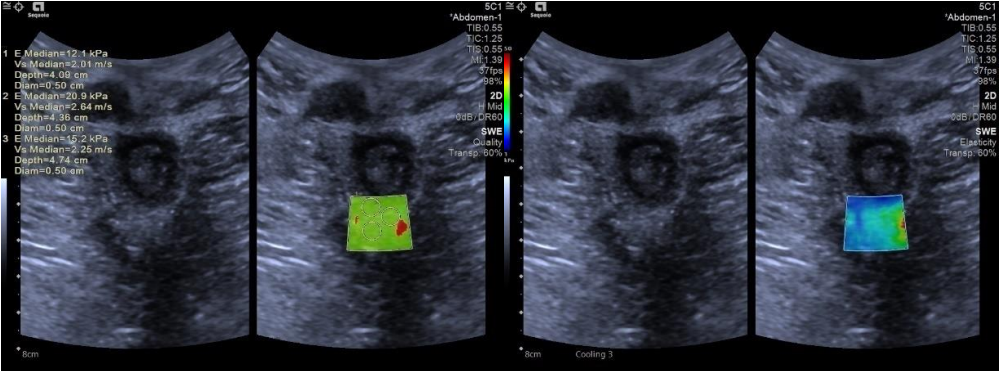


Fig 1

②Young's modulus value in the contracted state: The patient maintained maximum contraction of the pelvic floor muscles (with the contraction duration ≥ 3 seconds). The operator selected the "VT" function, placed the region of interest on the image of the pelvic floor muscles (keeping the size consistent with that in the resting state measurement), and clicked "Update" to generate an elastogram. Three positions within the region were selected for measurement.

**3.3 Pain scale (Visual Analogue Scale)**

The Visual Analogue Scale (VAS) is a commonly used unidimensional tool for assessing pain intensity. The core component of this scale is a 100-mm-long straight line, with one end marked as "no pain" and the other end marked as "extreme pain" or "the most intense pain imaginable". At the initiation and conclusion of treatment, patients are asked to mark a point (such as a dot or "✕") on this line to reflect their perceived pain intensity at that time. In this way, changes in the patient's pain level can be objectively evaluated.

**4. Withdrawal and Termination Criteria**

**4.1 Subject Withdrawal**

**4.1.1 Researcher-Decided Withdrawal**

Subjects will be withdrawn if:

1. Severe adverse events occur, precluding continued participation;

2. Subjects withdraw voluntarily;

3. Other circumstances render the subject unsuitable for the study

**4.1.2 Subject-Initiated Withdrawal**

Subjects have the right to withdraw at any time per the informed consent form. "Withdrawal" includes unplanned loss to follow-up. Researchers should document withdrawal reasons (e.g., inability to continue due to scheduling).

**4.1.3 Data Retention for Withdrawn**

Subjects All records of withdrawn subjects, including outcome indicators, will be retained.

**4.2 Study Termination**

The entire study may be terminated prematurely if:

① Severe unexpected adverse events (e.g., severe allergies, symptom exacerbation) occur;

② Subjects develop serious complications or rapidly deteriorating conditions;

③ Critical flaws in the study design or implementation compromise data validity. Termination must be promptly reported to all relevant parties (subjects, ethics committee, institutions).

**5. Data Management and Traceability**

**5.1 Data Management**

**5.1.1 Case Report Forms (CRFs)**

CRFs for all enrolled subjects are the property of the sponsor and may not be disclosed to third parties without authorization.

**5.1.2 Database Construction**

① Double data entry will be performed using independent software, with automatic error-checking and corrections.

② Queries on ambiguous data will be forwarded to researchers via clinical monitors for verification.

③ A 10% random sample of CRFs will undergo manual review for accuracy.

④ The database will be locked by the principal investigator, data manager, and statistician after confirming correctness, with no subsequent modifications allowed.

**5.2 Statistical Analysis**

The analysis was conducted using SPSS (Version 27). Data normality was assessed through visual inspection and the Shapiro-Wilk test. Continuous variables were reported as mean (SD) or median (first quartile [Q1]−third quartile [Q3]). The Student’s t-test or Mann–Whitney U test was employed to compare baseline characteristics and outcomes including VAS, s-EMG, thickness and Young's modulus of PFM between the two groups before and after the intervention. Within-group comparisons were performed using the paired t-test. *P* < 0.05 was considered statistically significant across the entire statistical analysis.

**6. Safety inspection**

For any unexpected events or adverse events occurring during the study period, the record form shall be filled out truthfully, including details of their manifestations, occurrence time, severity, duration, measures taken, and outcomes.

**7. Quality control measures**

**7.1 Quality control for subject inclusion**

①Subjects must strictly meet the inclusion criteria and not meet the exclusion criteria.

②Before inclusion, subjects should be informed of the possible discomfort that may occur during the experiment.

**7.2 Quality control of the treatment process**

① To prevent the occurrence of adverse reactions, patients should be informed of the general situation of the VR scenes before the formal intervention, and they should be allowed to adapt to the VR scenes first before proceeding with the formal training.

② To ensure the therapeutic effect, patients are instructed not to close their eyes during the VR pelvic floor relaxation training.

③ Patients are advised not to receive other treatments during the therapeutic period; those who are found to have received other treatments will be excluded.

**7.3 Quality control of evaluation methods**

① Researcher training: All evaluators will receive centralized and unified training before the implementation of the study to ensure consistency in the collection of observation data and the understanding and mastery of scoring criteria.

② Research record forms should be filled out completely and accurately as required, with arbitrary alterations avoided. Observation records for the same subject should be completed by the same person as far as possible.

③ During Glazer surface electromyography assessment, the results may be affected by the anxiety and tension of patients during their visit. To prevent such states from interfering with the assessment results, necessary guidance and explanations on correct pelvic floor muscle contraction will be provided to patients before the test. Additionally, surface electromyography assessment of pelvic floor muscles will be performed before the initial treatment and on the day after the final treatment to minimize interference with raw data.

④ All pelvic floor ultrasound examinations will be conducted by the same operator using the same instrument before and after treatment. The examination site will be kept consistent for each assessment, and each indicator to be measured will be assessed three times, with the average value taken.

**7.4 Quality control of statistical analysis**

① Data entered into the computer database and original data will be cross-checked by two evaluators to avoid input errors.

② In principle, there should be no missing values in the research report forms; in particular, important indicators (major safety indicators) must be clearly filled in. Basic data such as gender, date of birth, and enrollment date must not be missing. For results measured as zero or unmeasurable during the trial, corresponding symbols should be used instead of leaving them blank, so as to distinguish them from missing values. If missing values occur, the original data should first be checked to determine whether there was an omission. If it is confirmed to be an omission, it should be supplemented. If there is no record in the original data and the missing value is an important safety-related indicator, the subject should be notified to recheck immediately to ensure their safety. If the missing value affects the judgment of results, the case will be excluded.

**7.5 Quality control of medical ethics issues**

After subjects have fully understood the protocol, significance, and potential benefits of this study, they will sign an informed consent form. Partial free examinations or subsidies will be provided to subjects to ensure their compliance with the study.

**8. Research Ethics**

This protocol must receive approval from the Ethics Committee of Fujian University of Traditional Chinese Medicine Affiliated Rehabilitation Hospital before implementation. The committee may approve, approve with modifications, reject, or suspend the study. All subjects will receive detailed information about the study (purpose, nature, potential benefits, risks) and provide voluntary written informed consent prior to enrollment, in compliance with the Declaration of Helsinki and Good Clinical Practice (GCP) guidelines. This translation adheres to the formal, structured style required for medical journal submissions, ensuring clarity, technical accuracy, and compliance with ethical and methodological standards.
